# Factors associated with death, hospitalization, resignation, and sick leave from work among patients with schizophrenia in Japan: a nested case–control study using a large claims database

**DOI:** 10.1186/s12888-023-05474-5

**Published:** 2024-01-03

**Authors:** Ken Inada, Yoshitaka Saito, Kenji Baba, Daisuke Fukui, Yuriko Masuda, Sachie Inoue, Takahiro Masuda

**Affiliations:** 1 Department of Psychiatry, Kitasato University School of Medicine, 1-15-1 Kitasato, Minami-Ku, Sagamihara, Kanagawa 252-0374 Japan; 2 Medical Science, Sumitomo Pharma Co., Ltd., Osaka, Japan; 3 Global Data Design Office, Sumitomo Pharma Co., Ltd., Tokyo, Japan; 4 CRECON Medical Assessment Inc, Tokyo, Japan

**Keywords:** Schizophrenia, Depression, Death, Hospitalization, Continuous employment, Japan, Claims database survey, Constipation

## Abstract

**Background:**

Premature mortality, frequent relapse that easily leads to hospitalization, and discontinuous employment are key challenges for the treatment of schizophrenia. We evaluated risk factors for important clinical outcomes (death, hospitalization, resignation, and sick leave from work) in patients with schizophrenia in Japan.

**Methods:**

A nested case–control study was conducted for patients with schizophrenia identified in a Japanese claims database. For each outcome, the case was matched with up to four controls of the same age, sex, index year, and enrollment status (employee or dependent family). Potential risk factors were defined by prescriptions or diagnoses within 3 months prior to or in the month of the event. The association among potential risk factors and each outcome was evaluated using multivariable conditional logistic regression analysis with stepwise variable selection.

**Results:**

The number of cases and eligible patients for each outcome were 144 and 38,451 (death), 1,520 and 35,225 (hospitalization), 811 and 18,770 (resignation), and 4,590 and 18,770 (sick leave), respectively. Depression was a risk factor for death (odds ratio [OR]: 1.92; 95% confidence interval [CI]: 1.12, 3.29), hospitalization (OR: 1.22; 95% CI: 1.05, 1.42), and sick leave from work (OR: 1.46; 95% CI: 1.36, 1.57). Other risk factors for death were hospitalization history, Charlson Comorbidity Index (CCI) score, and prescription for laxatives. Prescriptions for hypnotics, laxatives, and anticholinergics were risk factors for hospitalization. Prescriptions for hypnotics and anticholinergics were risk factors for resignation. CCI score, prescription for hypnotics, laxatives, and antidiabetics were risk factors for sick leave from work.

**Conclusions:**

Our findings suggest that depression and some physical symptoms, such as constipation and extrapyramidal symptoms, are risk factors for important clinical outcomes in patients with schizophrenia. Attention should be paid to both depression and physical symptoms for the treatment of schizophrenia.

**Supplementary Information:**

The online version contains supplementary material available at 10.1186/s12888-023-05474-5.

## Background

The lifetime prevalence of schizophrenia is estimated to be 0.48% worldwide [1] and 0.59% in Japan [2]. Patients with schizophrenia have diverse symptoms including hallucination and delusion (positive symptoms), inactivity and social withdrawal (negative symptoms), cognitive impairment, and depressive symptoms. These symptoms have a negative impact on patients’ lifestyle, increase their physical disease risk, limit their social functioning, and result in loss of work productivity.

Schizophrenia is associated with approximately 14.5 years of potential life loss compared to the general population [3]. Relapses in schizophrenia occur frequently, and about one-fifth of patients with schizophrenia are hospitalized in Japan [4]. Thus, the employment rate of patients with schizophrenia is very low compared to the general population [5]. A recent Japanese study reported that 48.9% of patients with schizophrenia do not have a job [2]. Furthermore, patients with schizophrenia experience difficulties sustaining a job, and 51% resign within one year [6, 7]. Thus, treatments of schizophrenia to reduce premature mortality, prevent relapse, and maintain social functioning and an independent social life are required [8]. Death, hospitalization, and continuous employment are important clinical outcomes for evaluating the treatment of schizophrenia, and clarifying the risk factors for these outcomes is key to improving the treatment approach.

Treatments of schizophrenia are mainly based on pharmacotherapy with antipsychotics to improve psychotic symptoms. Continuous antipsychotic treatment is known to be an important factor for lowering risk of death and hospitalization for patients with schizophrenia [8, 9]. On the other hand, other symptoms and impairments, such as cognitive impairment, depressive symptoms, and various physical symptoms, including drug-induced ones, may increase mortality risk and cause declines in social functioning [10–13].

Depression in schizophrenia is a critical symptom, and can occur at any phase throughout the course of schizophrenia illness – at the prodromal, first-episode, acute, and chronic stable phases [14]; its prevalence among patients with schizophrenia was estimated as 32.6% by a meta-analysis [15]. Depression in schizophrenia is reported to be a risk factor for suicide [11, 16], which is associated with more life-years loss than other causes of death in patients with schizophrenia [10]. It has been reported that depressed patients with schizophrenia are more likely to use relapse-related mental health services compared to non-depressed patients with schizophrenia, and improvement of depressive symptoms also tended to improve these problems [17]. Recently, a cross-sectional study in Japan reported that patients with schizophrenia with moderate or severe depressive symptoms had higher levels of work productivity loss than those with mild or no depressive symptoms [2]. Therefore, depression in schizophrenia is presumed to be a risk factor for death, hospitalization, and lack of continuous employment.

The risk factors for these important clinical outcomes remain unclear, and these outcomes depend on the medical and social environments in individual countries. Therefore, it is important to accumulate clinical evidence by utilizing real-world data in the targeted country. To our knowledge, there is no large-scale longitudinal study in Japan that evaluated potential risk factors, including depression, for death, hospitalization, and lack of continuous employment in patients with schizophrenia.

In this study, we evaluated risk factors for the important clinical outcomes of death, hospitalization, and lack of continuous employment in patients with schizophrenia using a large claims database in Japan.

## Methods

### Data source

A nested case–control study was conducted using a claims database provided by JMDC Inc., consisting of data from health insurance societies. The number of people enrolled since 2005 is approximately 15 million. Enrolled people are employees and their dependent families [18]. In this study, we used anonymized processed claims data of patients with schizophrenia from January 2005 to January 2022.

### Patients

The study population was defined as patients meeting the following criteria: (i) having a diagnosis of schizophrenia (ICD-10 code: F20), and (ii) having data at least 6 months prior to the month of the first diagnosis (the index month). To obtain employment status data, we excluded patients aged 60 years (the general retirement age in Japan) and older at the index month. We also excluded patients with the following comorbidities within 6 months prior to the index month to identify typical schizophrenia patients: (i) dementia, as defined by receiving a prescription (WHO-ATC code: N06D); (ii) neoplasm (ICD-10 code: C00-D48); and (iii) other psychiatric or neurodevelopmental diseases, such as bipolar affective disorder (ICD-10 code: F31), depressive episodes (ICD-10 code: F32), recurrent depressive disorders (ICD-10 code: F33), specific personality disorders (ICD-10 code: F60), mental retardation (ICD-10 code: F70–F79), or pervasive developmental disorders (ICD-10 code: F84).

Among the eligible patients, we established four cohorts to evaluate each outcome. The death cohort consisted of all eligible patients. The hospitalization cohort consisted of the eligible patients who were not admitted to a psychiatric ward within 6 months prior to or during the index month. The resignation and sick leave from work cohorts consisted only of employees (excluding dependent family members).

### Outcomes

Death, hospitalization, resignation, and sick leave (temporary leave from work due to sickness) were evaluated as important clinical outcomes. The last two outcomes served as surrogate endpoints for continuous employment. In the database, death and resignation were defined by records regarding the reasons for withdrawal from the private company’s health insurance. Hospitalization was defined as admission to a psychiatric ward. In Japan, there is a “injury and sickness allowance” scheme that is paid from the health insurance system when an insured person is unable to work for a long period due to injury or sickness. Therefore, we defined “sick leave” as having a medical certificate from a medical institution, which is necessary to apply for the allowance.

### Potential risk factors

To identify factors associated with the important clinical outcomes of schizophrenia, we examined the following factors based on their clinical importance and data availability in the claims database: depression, physical comorbidities using Charlson Comorbidity Index (CCI) [19, 20], hospitalization history (only for the death outcome), and prescriptions for hypnotics, laxatives, benzodiazepines, sedatives, anticholinergics, antidiabetics, antihypertensives, and antidyslipidemics. Details of the definitions are described in Supplementary material 1. Prescriptions for the medications were used as surrogates for the symptoms/diseases treated by them. Hospitalization history was defined as admission to a psychiatric ward within 6 months prior to or in the month of the event. The other potential risk factors were defined as a prescription/diagnosis record within 3 months prior to or in the month of the event.

### Statistical analysis

For each outcome cohort, patients who experienced an event were identified as cases. Each case was matched with up to four control patients who did not experience an event until the same time point as when the case experienced the event. Control patients were randomly selected from patients with the same age, sex, index year, and enrollment status (employee or dependent family) as the case. To identify patients with schizophrenia more precisely in the claims database, only patients who had a prescription for antipsychotics within 3 months prior to or in the month of the event were eligible for case and control.

Conditional logistic regression analyses were conducted with outcome (case/control) as the dependent variable and potential risk factors as independent variables. First, univariable analyses were conducted to examine the association among each factor and outcome. Then, considering clinical perspectives, factors included in multivariable analyses were determined. In multivariable analyses, variables were selected by the stepwise method. Odds ratio (OR) and 95% confidence interval (CI) were estimated for each factor. For the secondary analysis, multivariable analysis without stepwise selection was also performed.

*P* < 0.05 was considered significant. All statistical analyses were performed with SAS ver. 9.4.

## Results

### Patients

**Fig. 1:**
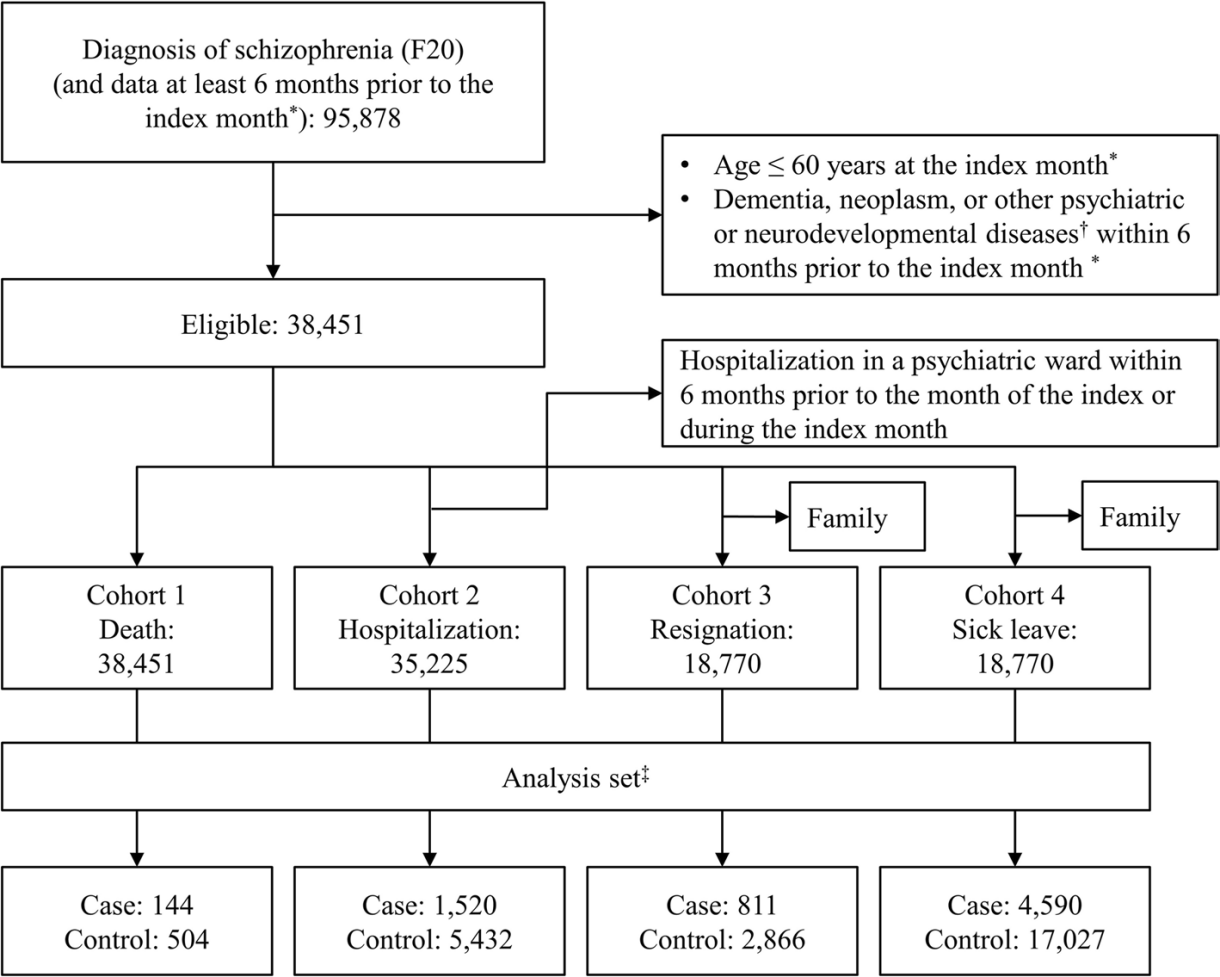
Flowchart of study population. *The first diagnosis month of schizophrenia. †Bipolar affective disorder (F31), depressive episodes (F32), recurrent depressive disorders (F33), specific personality disorders (F60), mental reatrdations (F70–F79), and pervasive developmental disorders (F84). ‡Patients with a prescription for an antipsychotic drug within 3 months prior to or in the month of the event

A total of 38,451 patients were identified as eligible patients (Fig. 1). All the eligible patients were included in the death cohort (*n* = 38,451), patients without hospitalization history to a psychiatric ward within 6 months prior to or in the index month were included in the hospitalization cohort (*n* = 35,225), and 18,770 employees were included in both the resignation and the sick leave cohorts.

The number of identified cases and controls were 144 and 504 in the death cohort, 1,520 and 5,432 in the hospitalization cohort, 811 and 2,866 in the resignation cohort, and 4,590 and 17,027 in the sick leave cohort, respectively. A summary of patient characteristics and the proportion of those with potential risk factors for the cases and controls are described in Table 1.

**Table 1:** Baseline characteristics and risk factors

**(a) Death**		
	**Case** **(*n* = 144)**	**Control** **(*n* = 504)**
Baseline characteristics		
Age (years), mean ± SD	42.0 ± 14.0	42.0 ± 13.7
Sex (male)	87 (60.4%)	311 (61.7%)
Follow-up period (month), median (IQR)	6.0 (3.0, 20.5)	5.0 (3.0, 15.0)
Risk factors		
Hospitalization (in 6 months)	24 (16.7%)	55 (10.9%)
Depression	43 (29.9%)	116 (23.0%)
CCI score, mean ± SD	2.3 ± 3.0	0.6 ± 1.2
Hypnotics prescription	93 (64.6%)	252 (50.0%)
Benzodiazepines prescription	98 (68.1%)	299 (59.3%)
Sedatives prescription	108 (75.0%)	324 (64.3%)
Laxatives prescription	60 (41.7%)	73 (14.5%)
Anticholinergics prescription	19 (13.2%)	60 (11.9%)
Antidiabetics prescription	17 (11.8%)	33 (6.5%)
Antihypertensives prescription	46 (31.9%)	90 (17.9%)
Antidyslipidemics prescription	8 (5.6%)	56 (11.1%)
**(b) Hospitalization**		
	**Case** **(*n* = 1,520)**	**Control** **(*n* = 5,432)**
Baseline characteristics		
Age (years), mean ± SD	29.2 ± 14.6	28.9 ± 14.7
Sex (male)	636 (41.8%)	2278 (41.9%)
Follow-up period (month), median (IQR)	6.0 (3.0, 19.0)	5.0 (2.0, 15.0)
Risk factors		
Depression	433 (28.5%)	1179 (21.7%)
CCI score, mean ± SD	0.4 ± 0.9	0.3 ± 0.8
Hypnotics prescription	1093 (71.9%)	2144 (39.5%)
Benzodiazepines prescription	1148 (75.5%)	2769 (51.0%)
Sedatives prescription	1227 (80.7%)	3035 (55.9%)
Laxatives prescription	452 (29.7%)	358 (6.6%)
Anticholinergics prescription	368 (24.2%)	524 (9.6%)
Antidiabetics prescription	35 (2.3%)	103 (1.9%)
Antihypertensives prescription	161 (10.6%)	406 (7.5%)
Antidyslipidemics prescription	67 (4.4%)	224 (4.1%)
**(c) Resignation**		
	**Case** **(*n* = 811)**	**Control** **(*n* = 2,866)**
Baseline characteristics		
Age (years), mean ± SD	33.6 ± 10.3	33.5 ± 10.0
Sex (male)	563 (69.4%)	2060 (71.9%)
Follow-up period (month), median (IQR)	5.0 (3.0, 15.0)	5.0 (2.0, 12.0)
Risk factors		
Depression	238 (29.3%)	860 (30.0%)
CCI score, mean ± SD	0.4 ± 0.9	0.3 ± 0.8
Hypnotics prescription	455 (56.1%)	1424 (49.7%)
Benzodiazepines prescription	542 (66.8%)	1807 (63.1%)
Sedatives prescription	567 (69.9%)	1916 (66.9%)
Laxatives prescription	73 (9.0%)	206 (7.2%)
Anticholinergics prescription	120 (14.8%)	289 (10.1%)
Antidiabetics prescription	23 (2.8%)	63 (2.2%)
Antihypertensives prescription	66 (8.1%)	210 (7.3%)
Antidyslipidemics prescription	38 (4.7%)	143 (5.0%)
**(d) Sick leave from work**		
	**Case** **(*n* = 4,590)**	**Control** **(*n* = 17,027)**
Baseline characteristics		
Age (years), mean ± SD	36.3 ± 10.8	36.3 ± 10.8
Sex (male)	3227 (70.3%)	12,094 (71.0%)
Follow-up period (month), median (IQR)	3.0 (2.0, 5.0)	3.0 (2.0, 5.0)
Risk factors		
Depression	1733 (37.8%)	4776 (28.1%)
CCI score, mean ± SD	0.6 ± 1.2	0.4 ± 0.9
Hypnotics prescription	3083 (67.2%)	8100 (47.6%)
Benzodiazepines prescription	3450 (75.2%)	10,401 (61.1%)
Sedatives prescription	3673 (80.0%)	11,160 (65.5%)
Laxatives prescription	713 (15.5%)	1295 (7.6%)
Anticholinergics prescription	541 (11.8%)	1581 (9.3%)
Antidiabetics prescription	199 (4.3%)	440 (2.6%)
Antihypertensives prescription	634 (13.8%)	1796 (10.5%)
Antidyslipidemics prescription	282 (6.1%)	958 (5.6%)

Data are presented as number (%) of patients unless otherwise indicated

*CCI* Charlson Comorbidities Index, *IQR* Interquartile range, *SD* Standard deviation

### Analysis results

#### Univariable analysis

The results of univariable analysis for each outcome are presented in Supplementary material 2.

#### Multivariable analysis

The factors of prescription for benzodiazepines and sedatives were excluded from the multivariable analysis because their definitions partly overlap with those of hypnotics, and the prescription for hypnotics is easier to interpret as a surrogate of sleep disturbance. The results of multivariable analysis are presented in Table 2. For death, we found hospitalization history, depression, CCI score, and prescription for laxatives as risk factors. On the other hand, prescription for antidyslipidemics lowered the risk. For hospitalization, we found depression, prescription for hypnotics, laxatives, and anticholinergics as risk factors. For resignation, we found prescription for hypnotics and anticholinergics as risk factors. For sick leave from work, we found depression, CCI score, and prescription for hypnotics, laxatives, and antidiabetics as risk factors.

**Table 2:** Results of logistic regression analysis (stepwise selection)

**(a) Death**			
Factor	**OR**	**95% CI**	***p* value**
Hospitalization history (in 6 months)	2.93	1.54, 5.59	< 0.01
Depression	1.92	1.12, 3.29	0.02
CCI score	1.63	1.39, 1.91	< 0.01
Laxatives prescription	2.60	1.42, 4.77	< 0.01
Antidyslipidemics prescription	0.24	0.10, 0.60	< 0.01
**(b) Hospitalization**			
Factor	**OR**	**95% CI**	***p* value**
Depression	1.22	1.05, 1.42	0.01
Hypnotics prescription	3.29	2.85, 3.79	< 0.01
Laxatives prescription	4.73	3.95, 5.67	< 0.01
Anticholinergics prescription	2.20	1.85, 2.61	< 0.01
**(c) Resignation**			
Factor	**OR**	**95% CI**	***p* value**
Hypnotics prescription	1.26	1.07, 1.48	0.01
Anticholinergics prescription	1.41	1.12, 1.78	< 0.01
**(d) Sick leave from work**			
Factor	**OR**	**95% CI**	***p* value**
Depression	1.46	1.36, 1.57	< 0.01
CCI score	1.11	1.07, 1.15	< 0.01
Hypnotics prescription	2.07	1.93, 2.23	< 0.01
Laxatives prescription	1.88	1.68, 2.09	< 0.01
Antidiabetics prescription	1.25	1.03, 1.53	0.03

*CCI* Charlson Comorbidities Index, *CI* Confidence interval, *OR* Odds ratio

The results are similar in the secondary analysis without stepwise selection (Supplementary material 3).

## Discussion

In this study, risk factors for the important clinical outcomes of patients with schizophrenia were evaluated by nested case–control design using a large-scale, health insurance-based database in Japan. Depression was identified as a risk factor for death, hospitalization, and sick leave. CCI score and prescription for laxatives were identified as risk factors for death and sick leave, indicating that physical comorbidities and constipation are risk factors for these important clinical outcomes. Constipation was also found as a risk factor for hospitalization, and extrapyramidal symptoms (EPS), indicated by anticholinergics prescription, was determined as a risk factor for hospitalization and resignation.

In this study, the proportions of patients with depression in the death, hospitalization, resignation, and sick leave cohorts were 24.5%, 23.2%, 29.9%, and 30.1%, respectively. The proportions were similar to a previous report that evaluated the prevalence of depression by meta-analysis [15]. As the study did not include Japanese data, this suggests that depression among patients with schizophrenia is a common symptom worldwide. A previous study reported that depression is a critical symptom of schizophrenia and a risk factor for suicide [11]. Suicide is associated with many life-years loss, but it is not the major cause of death in patients with schizophrenia [10, 13, 21]; therefore, it is notable that depression for patients with schizophrenia was identified to be a risk factor for all-cause death, including suicide, in this study. This finding suggests that depression affects deaths caused by accidents and physical disease, as depression lowers the self-management abilities of patients with schizophrenia and leads to an unhealthy lifestyle. In our study, hypertension and diabetes, which are well-known to lead to cardiovascular events, were not detected as risk factors for death, which may be because the eligible patients in our study were under 60 years old. On the other hand, our results indicated that dyslipidemia was associated with a reduced risk of death. This finding was unexpected, as dyslipidemia is also known to be a risk factor for cardiovascular events. Further studies are needed to confirm this result.

Identified risk factors for hospitalization to a psychiatric ward were not only psychiatric conditions, such as depression and sleep disturbance, but also physical conditions such as constipation and EPS. Our findings support a previous study that depression increases the risk of hospitalization and rehospitalization in patients with schizophrenia [17] and a previous systematic review that reported that sleep disturbance was associated with positive symptoms and poor clinical outcome [22]. EPS and constipation are common side effects of antipsychotic medication. In clinical practice, patients who experience side effects are required to reduce dosage or switch to other antipsychotics [23]. Therefore, patients with EPS or constipation may have difficulty managing psychotic symptoms. EPS induces and overlaps with depressive symptoms [24], and constipation is common in depressed individuals [25]. Moreover, sleep disturbance is strongly associated with depression [26]. Thus, depression may be a key risk factor for hospitalization, although these are independent risk factors. Lifestyle diseases, such as hypertension and diabetes, were not identified as risk factors for hospitalization. The results are clinically understandable because patients with schizophrenia are unlikely to be hospitalized to a psychiatric ward due to lifestyle diseases in Japan.

Cognitive function impairment, negative symptoms, and education were reported to be predictors of employment status in patients with schizophrenia [27]. However, few studies have examined risk factors in patients with schizophrenia for continuous employment-related outcomes such as sick leave and resignation. A case–control study in Germany reported that schizophrenia severity, assistance or care in everyday life, age, and typical antipsychotic treatment were associated with early retirement in patients with schizophrenia; however, depression was not included as a candidate risk factor in the analysis [28]. Our study found that depression was not associated with resignation, but was a significant risk factor for sick leave from work. To our knowledge, this is the first study to identify depression as a risk factor for sick leave in patients with schizophrenia. However, the results were unexpected because we hypothesized that depression would be both a risk factor for resignation and sick leave. There may be differences of the characteristics between patients who took sick leave and those who resigned. Although we did not evaluate duplicate cases in sick leave and resignation, the ratio of depression was higher in cases of sick leave than resignation (37.8% vs. 29.3%), and the number of the resignation cases (*n* = 811) was only about one-fifth of the sick leave cases (*n* = 4,590). Similar to our results, a cohort study composed of employees in 11 Japanese large private companies reported that among the employees with schizophrenia who had sick leave, the resignation rate was only 28.6% within 3.5 years and 66.7% returned to work [29]. In the Netherlands, which has a different medical and social environment from Japan, 76% of employees taking sick leave due to schizophrenia returned to work within 2 years [30]. Thus, most employees who take sick leave due to schizophrenia are able to return to work. These findings may explain why depression was a risk factor for sick leave but not resignation in the present study. We also identified EPS as a risk factor for resignation. The result is similar to the previous German case–control study that reported that the proportion of patients with EPS was about twice that among patients who took early retirement compared to those that did not [28].

Medical and social environments are markedly differ among countries, and are thought to affect our focusing outcomes, especially employment-related outcomes. Thus, in this study, we investigated risk factors in patients with schizophrenia for resignation and sick leave from work using a Japanese database, and found that depression was a risk factor for sick leave. Furthermore, sleep disturbance, constipation, and physical comorbidities (CCI score), which may be related to depression, were indicated to be risk factors for sick leave.

Since this study is based on medical claims data, there are several limitations due to data availability and characteristics. The first limitation is the definition of resignation. We defined resignation based on the reason for withdrawal from the private company’s health insurance. Thus, patients who quit the company and work in another company may have been misclassified, which may have led to underestimating the association of potential risk factors. This may explain why depression and constipation were not associated with resignation despite being strong risk factors for the other three outcomes. Second, we could not evaluate the following factors, which are thought to affect the outcomes that we investigated: social environmental factors, such as continuous employment support service, employment systems, and living alone, and lifestyle factors, such as physical activity and eating habits; and psychotherapy or counseling intervention. Third, since adherence to antipsychotics was suggested to be a strong risk factor for death, hospitalization, and early retirement [8, 9, 28], we included only people with a diagnosis of schizophrenia and prescription data for antipsychotics as case or control, but we could not evaluate their medication adherence status due to data characteristics, and we did not focus on the details of antipsychotics, such as type, dosage, and the number of antipsychotics prescribed to the patients. Fourth, we could not distinguish detailed depressive features, such as post-psychotic depression and depressed symptoms due to negative symptoms in patients with schizophrenia.

## Conclusions

This study suggests that depression in patients with schizophrenia is a risk factor for important clinical outcomes such as death, hospitalization, and sick leave from work. Also, physical conditions, such as constipation and EPS, are risk factors for the important clinical outcomes that we investigated. These findings suggest that healthcare professionals should carefully pay attention to both psychiatric symptoms and physical symptoms for the treatment of patients with schizophrenia.

### Supplementary Information

**Additional file 1:** **Supplementary material 1.** Definitions of risk factors. **Supplementary material 2.** Results of univariate logistic regression analysis. **Supplementary material 3.** Results of logistic regression analysis (without stepwise selection). 

## Data Availability

The datasets generated and/or analyzed during the present study are not publicly available because they were purchased from a commercial provider (JMDC Inc.); however, they are available from the corresponding author on reasonable request.

## Abbreviations

CCI: Charlson comorbidity index
CI: Confidence interval
EPS: Extrapyramidal symptom
IQR: Interquartile range
OR: Odds ratio
SD: Standard deviation
