# Supplementary material for: Factors associated with death, hospitalization, resignation, and sick leave from work among patients with schizophrenia in Japan: a nested case–control study using a large claims database
Source: BMC Psychiatry. 2024 Jan 3;24:22. doi: 10.1186/s12888-023-05474-5 (PMC10765934; doi:10.1186/s12888-023-05474-5)
Supplement: Supplementary file 1 — Additional file 1: Supplementary material 1. Definitions of risk factors. Supplementary material 2. Results of univariate logistic regression analysis. Supplementary material 3. Results of logistic regression analysis (without stepwise selection). [file 12888_2023_5474_MOESM1_ESM.docx]

Research article

**Factors associated with death, hospitalization, resignation and sick leave from work among patients with schizophrenia in Japan: A nested case-control study using a large claims database**

**Author:**

Ken Inada^1^, Yoshitaka Saito^1^, Kenji Baba^2^, Daisuke Fukui^2^, Yuriko Masuda^3^, Sachie Inoue^3^, Takahiro Masuda^2^

1 Department of Psychiatry, Kitasato University School of Medicine

2 Sumitomo Pharma Co. Ltd.

3 CRECON Medical Assessment Inc.

**Corresponding author:**

Ken Inada, M.D., Ph.D.

Department of Psychiatry, Kitasato University School of Medicine

1-15-1 Kitasato, Minami-ku, Sagamihara, Kanagawa, Japan, 252-0374

Tel +81-42-778-8111

E-mail: inadaken@kitasato-u.ac.jp

**Supplementary material 1.** **Definitions of risk factors**

(a) List of risk factors

| Factor | Definition detail |
| --- | --- |
| Hospitalization  (in 6 months) | Receipt of hospitalization fee for psychiatric ward |
| Depression | Diagnosis of depression (ICD-10 code: F32, F33) and prescription of antidepressant (drugs listed in Supplementary material 1b; any formulation) |
| CCI score | Calculated score suggested by Charlson, 1987 [1]. Definition of each comorbidity is based on Quan, 2005 [2]. |
| Hypnotics prescription | Prescription of oral drugs listed in Supplementary material 1b |
| Benzodiazepines prescription | Prescription of oral drugs listed in Supplementary material 1b |
| Sedatives prescription | Prescription of oral drugs listed in Supplementary material 1b |
| Laxatives prescription | Prescription of laxative (WHO-ATC code: A06A, any formulation) |
| Anticholinergics prescription | Prescription of drugs listed in Supplementary material 1b; any formulation |
| Antidiabetics prescription | Prescription of antidiabetics (WHO-ATC code: A10; any formulation) |
| Antihypertensives prescription | Prescription of antihypertensives (WHO-ATC code:C02,03,07-09,11; any formulation) |
| Antidyslipidemics prescription | Prescription of antidyslipidemics (WHO-ATC code: C10; any formulation) |

(b) Drugs defined by generic names

| Drug | Generic name | Formulation |
| --- | --- | --- |
| Antidepressants | Imipramine | Any formulation |
|  | Clomipramine |  |
|  | Trimipramine |  |
|  | Lofepramine |  |
|  | Amitriptyline |  |
|  | Nortriptyline |  |
|  | Dosulepin |  |
|  | Amoxapine |  |
|  | Maprotiline |  |
|  | Paroxetine |  |
|  | Sertraline |  |
|  | Fluvoxamine |  |
|  | Escitalopram |  |
|  | Setiptiline |  |
|  | Mianserin |  |
|  | Trazodone |  |
|  | Mirtazapine |  |
|  | Venlafaxine |  |
|  | Milnacipran |  |
|  | Duloxetine |  |
|  | Vortioxetine |  |
| Benzodiazepines | Clonazepam | Oral |
|  | Diazepam |  |
|  | Chlordiazepoxide |  |
|  | Medazepam |  |
|  | Potassium clorazepate |  |
|  | Lorazepam |  |
|  | Bromazepam |  |
|  | Clobazam |  |
|  | Prazepam |  |
|  | Alprazolam |  |
|  | Fludiazepam |  |
|  | Ethyl loflazepate |  |
|  | Etizolam |  |
|  | Clotiazepam |  |
|  | Cloxazolam |  |
|  | Tofisopam |  |
|  | Mexazolam |  |
|  | Haloxazolam |  |
|  | Rilmazafone |  |
|  | Flurazepam |  |
|  | Nitrazepam |  |
|  | Flunitrazepam |  |
|  | Estazolam |  |
|  | Triazolam |  |
|  | Lormetazepam |  |
|  | Midazolam |  |
|  | Brotizolam |  |
|  | Quazepam |  |
|  | Remimazolam |  |
|  | Nimetazepam |  |
|  | Zopiclone |  |
|  | Zolpidem |  |
|  | Eszopiclone |  |
| Hypnotics | Phenobarbital | Oral |
|  | Clonazepam |  |
|  | Chlorpromazine hydrochloride/Promethazine hydrochloride/Phenobarbital |  |
|  | Flutoprazepam |  |
|  | Oxazolam |  |
|  | Haloxazolam |  |
|  | Rilmazafone |  |
|  | Flurazepam |  |
|  | Nitrazepam |  |
|  | Flunitrazepam |  |
|  | Estazolam |  |
|  | Triazolam |  |
|  | Lormetazepam |  |
|  | Brotizolam |  |
|  | Quazepam |  |
|  | Nimetazepam |  |
|  | Zopiclone |  |
|  | Zolpidem |  |
|  | Eszopiclone |  |
|  | Melatonin |  |
|  | Ramelteon |  |
|  | Bromisoval |  |
|  | Triclofos |  |
|  | Suvorexant |  |
|  | Lemborexant |  |
| Sedatives | Phenobarbital | Oral |
|  | Clonazepam |  |
|  | Chlorpromazine hydrochloride/Promethazine hydrochloride/Phenobarbital |  |
|  | Flutazolam |  |
|  | Flutoprazepam |  |
|  | Oxazolam |  |
|  | Diazepam |  |
|  | Chlordiazepoxide |  |
|  | Medazepam |  |
|  | Potassium clorazepate |  |
|  | Lorazepam |  |
|  | Bromazepam |  |
|  | Prazepam |  |
|  | Alprazolam |  |
|  | Fludiazepam |  |
|  | Ethyl loflazepate |  |
|  | Etizolam |  |
|  | Clotiazepam |  |
|  | Cloxazolam |  |
|  | Tofisopam |  |
|  | Benzodiazepine derivatives |  |
|  | Hydroxyzine |  |
|  | Tandospirone |  |
|  | Pentobarbital |  |
|  | Amobarbital |  |
|  | Barbital |  |
|  | Chloral hydrate |  |
|  | Haloxazolam |  |
|  | Rilmazafone |  |
|  | Flurazepam |  |
|  | Nitrazepam |  |
|  | Flunitrazepam |  |
|  | Estazolam |  |
|  | Triazolam |  |
|  | Lormetazepam |  |
|  | Brotizolam |  |
|  | Quazepam |  |
|  | Nimetazepam |  |
|  | Zopiclone |  |
|  | Zolpidem |  |
|  | Eszopiclone |  |
|  | Melatonin |  |
|  | Ramelteon |  |
|  | Bromisoval |  |
|  | Triclofos |  |
|  | Suvorexant |  |
|  | Lemborexant |  |
| Anticholinergics | Trihexyphenidyl | Any formulation |
|  | Biperiden |  |
|  | Procyclidine |  |
|  | Mazaticol |  |
|  | Benztropine |  |
|  | Amantadine |  |
|  | Promethazine |  |
| Antipsychotic | Chlorpromazine | Any formulation |
|  | Levomepromazine |  |
|  | Fluphenazine |  |
|  | Perphenazine |  |
|  | Prochlorperazine |  |
|  | Trifluoperazine |  |
|  | Periciazine |  |
|  | Thioridazine |  |
|  | Spiperone |  |
|  | Timiperone |  |
|  | Haloperidol |  |
|  | Moperone |  |
|  | Pipamperone |  |
|  | Bromperidol |  |
|  | Oxypertine |  |
|  | Lurasidone |  |
|  | Pimozide |  |
|  | Clozapine |  |
|  | Olanzapine |  |
|  | Quetiapine |  |
|  | Asenapine |  |
|  | Benzamides |  |
|  | Sulpiride |  |
|  | Sultopride |  |
|  | Tiapride |  |
|  | Blonanserin |  |
|  | Carpipramine |  |
|  | Clocapramine |  |
|  | Perospirone |  |
|  | Risperidone |  |
|  | Mosapramine |  |
|  | Zotepine |  |
|  | Aripiprazole |  |
|  | Paliperidone |  |
|  | Brexpiprazole |  |
|  | Reserpine |  |

CCI, Charlson Comorbidities Index.

**Supplementary material 2. Results of univariate logistic regression analysis**

(a) Death

| Variable | OR | 95% CI | p value |
| --- | --- | --- | --- |
| Hospitalization (in 6 months) | 1.77 | 1.04, 3.00 | 0.04 |
| Depression | 1.50 | 0.98, 2.29 | 0.06 |
| CCI score | 1.62 | 1.42, 1.84 | <0.01 |
| Hypnotics prescription | 1.76 | 1.18, 2.64 | 0.01 |
| Benzodiazepines prescription | 1.47 | 0.97, 2.20 | 0.07 |
| Sedatives prescription | 1.66 | 1.08, 2.57 | 0.02 |
| Laxatives prescription | 5.49 | 3.34, 9.03 | <0.01 |
| Anticholinergics prescription | 1.13 | 0.65, 1.98 | 0.67 |
| Antidiabetics prescription | 1.75 | 0.95, 3.25 | 0.07 |
| Antihypertensives prescription | 2.47 | 1.54, 3.95 | <0.01 |
| Antidyslipidemics prescription | 0.40 | 0.19, 0.88 | 0.02 |

(b) Hospitalization

| Variable | OR | 95% CI | p value |
| --- | --- | --- | --- |
| Depression | 1.45 | 1.27, 1.66 | <0.01 |
| CCI score | 1.09 | 1.02, 1.17 | 0.01 |
| Hypnotics prescription | 4.26 | 3.72, 4.87 | <0.01 |
| Benzodiazepines prescription | 3.38 | 2.94, 3.89 | <0.01 |
| Sedatives prescription | 3.63 | 3.13, 4.21 | <0.01 |
| Laxatives prescription | 6.49 | 5.48, 7.68 | <0.01 |
| Anticholinergics prescription | 2.98 | 2.55, 3.48 | <0.01 |
| Antidiabetics prescription | 1.12 | 0.74, 1.70 | 0.59 |
| Antihypertensives prescription | 1.61 | 1.30, 1.98 | <0.01 |
| Antidyslipidemics prescription | 1.03 | 0.76, 1.39 | 0.87 |

(c) Resignation

| Variable | OR | 95% CI | p value |
| --- | --- | --- | --- |
| Depression | 0.99 | 0.83, 1.18 | 0.91 |
| CCI score | 1.05 | 0.95, 1.15 | 0.36 |
| Hypnotics prescription | 1.30 | 1.10, 1.52 | <0.01 |
| Benzodiazepines prescription | 1.17 | 0.99, 1.39 | 0.06 |
| Sedatives prescription | 1.15 | 0.97, 1.37 | 0.10 |
| Laxatives prescription | 1.26 | 0.95, 1.69 | 0.12 |
| Anticholinergics prescription | 1.48 | 1.17, 1.86 | <0.01 |
| Antidiabetics prescription | 1.26 | 0.77, 2.08 | 0.36 |
| Antihypertensives prescription | 1.18 | 0.86, 1.62 | 0.31 |
| Antidyslipidemics prescription | 0.87 | 0.59, 1.27 | 0.47 |

(d) Sick leave from work

| Variable | OR | 95% CI | p value |
| --- | --- | --- | --- |
| Depression | 1.55 | 1.45, 1.66 | <0.01 |
| CCI score | 1.20 | 1.16, 1.24 | <0.01 |
| Hypnotics prescription | 2.29 | 2.14, 2.46 | <0.01 |
| Benzodiazepines prescription | 1.92 | 1.78, 2.06 | <0.01 |
| Sedatives prescription | 2.10 | 1.94, 2.28 | <0.01 |
| Laxatives prescription | 2.30 | 2.08, 2.55 | <0.01 |
| Anticholinergics prescription | 1.27 | 1.15, 1.41 | <0.01 |
| Antidiabetics prescription | 1.76 | 1.47, 2.10 | <0.01 |
| Antihypertensives prescription | 1.43 | 1.29, 1.59 | <0.01 |
| Antidyslipidemics prescription | 1.09 | 0.94, 1.26 | 0.26 |

CCI, Charlson Comorbidities Index; CI, confidence interval; OR, odds ratio.

**Supplementary material 3. Results of logistic regression analysis (without stepwise selection)**

(a) Death

| Variable | OR | 95% CI | p value |
| --- | --- | --- | --- |
| Hospitalization (in 6 months) | 2.95 | 1.52, 5.75 | <0.01 |
| Depression | 1.88 | 1.09, 3.24 | 0.02 |
| CCI score | 1.58 | 1.35, 1.86 | <0.01 |
| Hypnotics prescription | 1.20 | 0.73, 1.96 | 0.47 |
| Laxatives prescription | 2.48 | 1.33, 4.63 | <0.01 |
| Anticholinergics prescription | 0.92 | 0.46, 1.85 | 0.81 |
| Antidiabetics prescription | 1.36 | 0.55, 3.38 | 0.51 |
| Antihypertensives prescription | 1.26 | 0.63, 2.54 | 0.51 |
| Antidyslipidemics prescription | 0.20 | 0.07, 0.55 | <0.01 |

(b) Hospitalization

| Variable | OR | 95% CI | p value |
| --- | --- | --- | --- |
| Depression | 1.22 | 1.05, 1.42 | 0.01 |
| CCI score | 0.93 | 0.85, 1.01 | 0.08 |
| Hypnotics prescription | 3.29 | 2.86, 3.79 | <0.01 |
| Laxatives prescription | 4.85 | 4.03, 5.83 | <0.01 |
| Anticholinergics prescription | 2.19 | 1.84, 2.60 | <0.01 |
| Antidiabetics prescription | 0.81 | 0.50, 1.34 | 0.42 |
| Antihypertensives prescription | 1.30 | 1.01, 1.67 | 0.04 |
| Antidyslipidemics prescription | 0.88 | 0.62, 1.26 | 0.48 |

(c) Resignation

| Variable | OR | 95% CI | p value |
| --- | --- | --- | --- |
| Depression | 0.96 | 0.80, 1.14 | 0.63 |
| CCI score | 1.02 | 0.92, 1.13 | 0.72 |
| Hypnotics prescription | 1.26 | 1.07, 1.49 | 0.01 |
| Laxatives prescription | 1.16 | 0.87, 1.57 | 0.32 |
| Anticholinergics prescription | 1.40 | 1.11, 1.77 | 0.01 |
| Antidiabetics prescription | 1.26 | 0.73, 2.19 | 0.41 |
| Antihypertensives prescription | 1.11 | 0.79, 1.57 | 0.54 |
| Antidyslipidemics prescription | 0.76 | 0.51, 1.14 | 0.19 |

(d) Sick leave from work

| Variable | OR | 95% CI | p value |
| --- | --- | --- | --- |
| Depression | 1.46 | 1.36, 1.57 | <0.01 |
| CCI score | 1.10 | 1.06, 1.15 | <0.01 |
| Hypnotics prescription | 2.06 | 1.91, 2.21 | <0.01 |
| Laxatives prescription | 1.86 | 1.67, 2.08 | <0.01 |
| Anticholinergics prescription | 1.06 | 0.95, 1.19 | 0.27 |
| Antidiabetics prescription | 1.26 | 1.03, 1.55 | 0.02 |
| Antihypertensives prescription | 1.11 | 0.99, 1.25 | 0.09 |
| Antidyslipidemics prescription | 0.89 | 0.76, 1.04 | 0.15 |

CCI, Charlson Comorbidities Index; CI, confidence interval; OR, odds ratio.

**References**

1. Charlson ME, Pompei P, Ales KL, MacKenzie CR. A new method of classifying prognostic comorbidity in longitudinal studies: development and validation. J Chronic Dis. 1987;40(5):373-83.
2. Quan H, Sundararajan V, Halfon P, Fong A, Burnand B, Luthi JC, et al. Coding algorithms for defining comorbidities in ICD-9-CM and ICD-10 administrative data. Med Care. 2005;43(11):1130-9.
